# Single-Crystal Diffraction at ORNL: Historical Development, Current Advances, and Future Perspectives

**DOI:** 10.1063/4.0000964

**Published:** 2025-10-27

**Authors:** Xiaoping Wang

**Affiliations:** Oak Ridge National Laboratory, Oak Ridge, Tennessee, USA

## Abstract

Since the pioneering neutron diffraction experiments by Ernest O. Wollan and Clifford G. Shull in the 1940s, Oak Ridge National Laboratory (ORNL) has played a central role in the advancement of single-crystal diffraction techniques. Over the past 75 years, ORNL's contributions have significantly shaped structural science across a range of disciplines, especially in chemistry, condense matter physics and materials science.

Among the suite of single-crystal diffractometers at the High Flux Isotope Reactor (HFIR) and the Spallation Neutron Source (SNS), the TOPAZ instrument plays a central role in extending neutron crystallography beyond traditional three-dimensional frameworks. By combining neutron wavelength-resolved Laue diffraction with event-based neutron detection, TOPAZ enables simultaneous measurement of three-dimensional diffraction space and real-time tracking of structural responses to external stimuli in parameter space—including temperature, pressure, and applied fields. These capabilities are particularly valuable for investigating hydrogen bonding in functional energy materials, local and short-range correlations in quantum materials, and structural and magnetic phase transitions under operando conditions.

Looking ahead, the integration of artificial intelligence (AI) and machine learning (ML) into neutron diffraction workflows is expected to enhance data interpretation, support real-time experimental optimization, and enable adaptive exploration of complex structural systems. These developments will broaden the scope of crystallographic research in structural science and create new opportunities for discovery in the study of advanced energy and quantum materials.

